# Design of Novel Human Wrist Prostheses Based on Parallel Architectures: Dimensional Synthesis and Kinetostatics

**DOI:** 10.3390/biomimetics10010044

**Published:** 2025-01-12

**Authors:** Raffaele Di Gregorio

**Affiliations:** Laboratory of Mechatronics and Virtual Prototyping (LaMaViP), Department of Engineering, University of Ferrara, Via Saragat 1, 44122 Ferrara, Italy; raffaele.digregorio@unife.it; Tel.: +39-0532-974828

**Keywords:** human wrist, prosthesis, parallel mechanism, dimensional synthesis, kinetostatics

## Abstract

The human wrist affects the ability to efficiently perform many manipulation tasks. Despite this, most upper-limb prostheses are focused on the hand’s mobility, which makes users compensate for the lost wrist mobility with complex manipulation strategies relying on the mobility of other body parts. In this context, research on wrist prostheses is still open to new contributions, even though a number of such prostheses are already present in the literature and on the market. In particular, the potential uses of parallel mechanisms in wrist prosthesis design have not been fully explored yet. In this work, after recalling the mobility characteristics of human wrists and reviewing the literature both on wrist prostheses and parallel mechanisms, a number of parallel architectures employable in a wrist prosthesis are selected. Then, with reference to the design requirements of this prosthesis type, the dimensional synthesis and kinetostatic analysis of the selected architectures are addressed. The results of this work are new wrist prosthesis architectures together with the analysis of their kinetostatic performances. These findings complete the first step of a research project aimed at developing new concepts for mechatronic wrists.

## 1. Introduction

The human wrist affects the ability to efficiently perform many manipulation tasks [1]. Compensating for the lost wrist mobility by means of movement of other body parts increases users’ perceived disability and may cause overuse, pain and even long-term damage to the involved body parts [2].

Despite this, most upper-limb prostheses are focused only/mainly on the hand’s mobility. Research on wrist prostheses is less developed than that on hand prostheses. Consequently, even though a number of such prostheses are already present in the literature and on the market [3,4,5], there is room for new analyses and ideas. For instance, the potential use of parallel mechanisms in wrist prosthesis design has not been fully explored yet.

**Figure 1:**
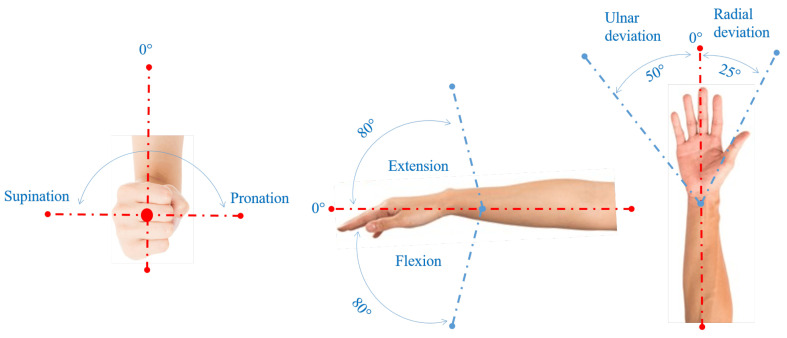
Hand’s DOFs due to wrist and forearm (reproduced from [6]).

From a kinematic point of view (see Figure 1 (reproduced from [6])), the human wrist [7,8] is a two-degrees-of-freedom (DOFs) mechanism that constrains the hand to perform a 2-DOF about-spherical motion (flexion/extension (FE) and radial/ulnar (RU) deviations) with respect to the forearm bones (radius and ulna). Also, the radius and ulna add a third rotation (pronation/supination (PS)) around the longitudinal axis of the forearm to the hand motion. Accordingly, a complete wrist prosthesis, which includes also the third rotation, should perform a 3-DOF about-spherical motion. Such a motion can be obtained through many different types of mechanisms.

The already presented wrist prostheses (WPs) are categorizable [4] according to their DOFs into single-DOF, two-DOF and three-DOF WPs. In each of these WP categories, they are further groupable into serial, parallel and hybrid architectures according to their kinematic architecture (topology) and into passive (i.e., externally moved by the user), body-powered (i.e., actuated through harnesses transmitting the motion of other body parts to the WP) and active (i.e., with motorized joints) WPs according to their actuation.

Single-DOF WPs are essentially revolute (R) pairs (see [4,9,10,11,12,13,14,15], for instance). They are named “flexors” (“rotators”) when the R-pair axis is perpendicular (parallel) to the forearm axis: that is, when they generate FE (PS) motion of the artificial hand. In some cases, they can be serially connected to generate two- and three-DOF WPs [16,17,18,19]; in particular, the serial combination rotator–flexor–rotator with a roll–pitch–yaw axis arrangement reproduces PS-FE-RU motions. Nevertheless, such dispositions are usually not able to match the weight and size requirements of WPs.

Ad hoc conceived two/three-DOF mechanisms for WPs (see [20,21,22,23,24,25,26,27,28] for instance) allow us to optimize specific features, e.g., the range of motion (RoM) or torque, etc., and obtain devices that are, in general, more compact than the modular multi-DOF WPs obtained by combining single-DOF WPs. Nevertheless, none of the already proposed WPs [4,5,24] are able to replicate all the performances of a human wrist. In particular, the size, weight and torque of a human wrist are the most difficult features to reproduce with a WP.

This work aims to investigate the potential of parallel architectures when used for ideating WPs. The adopted approach is systematic. In particular, after recalling some necessary background materials both on human wrist mobility/performances and possible parallel/hybrid architectures usable as WPs, with reference to WPs’ design requirements, the most promising of them are selected. Then, the dimensional synthesis of the selected WP architectures is addressed together with their kinetostatic analysis.

The result consists of the identification of four novel WP architectures: two with three DOFs that are based on novel types of parallel mechanisms (PMs) and two with two DOFs that are obtained by redesigning PM types previously proposed by the author for other applications. The proposed WPs have the following features: (i) they are all single-loop architectures with a reduced number of links whose DOFs are independently controllable, (ii) they cover all the mobility requirements by maintaining a compact overall size and (iii) they have good kinetostatic performances.

The paper is organized as follows. Section 2 provides the necessary background, selects the WP architectures and defines the design methodology. Then, Section 3 addresses the dimensional synthesis and the kinetostatic analysis of the selected architectures. Finally, Section 4 discusses the obtained results and Section 5 draws the conclusions.

## 2. Materials and Methods

The human wrist performances are analyzed in the literature [3,4,5,7,24] from the kinematics and the statics points of view.

Regarding wrist kinematics, the literature [3,4,5,7,24,29,30,31] synthesizes the data on human-wrist mobility by providing, for each DOF, the extremes of the *active range of motion* (AROM) and of the range of motion (RoM) required in the *activities of daily living* (ADLs) with reference to a neutral position (see Figure 1). In general, ADLs values are substantially (from 20% to 50% according to [5,7,29]) lower than the AROM, and the reported values depend on the analyzed sample of population. Damerla et al. [24], who cite an ample literature, concluded that the values reported in Table 1, which are the mean values of those reported in the cited literature, are believable references. Eventually, in most ADLs, RU could be replaced by small movements of shoulder and elbow joints practically without discomfort, which justifies the fact that many commercial 2-DOF WPs try to replicate only FE and PS.

Another relevant datum of wrist kinematics is the *joint speed* (JS). Unfortunately, studies aiming at providing reference JS values are less extended than those on RoM and are mainly focused on sports activities and athletes. Anyway, it is clear [24] that JS extreme values in ADLs are much smaller than JS peak values, which range from 10 rad/s to 38 rad/s according to the motion type [24], or JS extreme values in sports activities, which range from 3 rad/s to 19 rad/s according to the motion type [24]. Table 1 reports also JS in ADLs when available.

Regarding wrist statics, the literature [3,4,5,24] synthesizes the data on human wrist performances by providing, for each DOF, the extremes of the *joint torque* (JT). JT is measured for different wrist postures and/or motion conditions (i.e., different values of JS). The collected data vary according to the gender and the type of test. Damerla et al. [24] summarize the data reported in the literature in a table (Table IV of [24]) where, for men and women, the absolute maximum (i.e., the maximum values obtained by considering all the test types) and the mean maximum (i.e., the mean values of the maxima obtained in different test types) are given. In addition, they suggest that the mean maximum should be considered when designing a WP. Such mean maxima are reported in Table 1.

In prosthesis design, the ponderal and dimensional aspects must be also considered. Statistical studies on the weight of upper-limb parts are present in the literature. Nevertheless, data that are specifically related to the wrist weight are not reported in them, since the wrist parts are usually included in the hand and/or in the forearm when evaluating their weights. Such data have been summarized in [32] by providing the following mean values: (a) for men, the hand (the forearm) weighs 540 g (1420 g), which is the 0.63% (1.66%) of their body weight, and (b) for women, the hand (the forearm) weighs 380 g (1060 g), which is the 0.53% (1.48%) of their body weight. Consequently, for the WP design, it is reasonable to state that the overall weight of hand plus forearm, including the wrist, must not greatly change after the introduction of a WP with respect to that of the healthy limb.

Regarding the dimensional aspect, the mean values of the data that characterize the human wrist’s cross-section are reported in the literature and are summarized in [24] as follows (*wt*, *ww* and *wc* stand for *wrist thickness* (i.e., the smaller side of the circumscribed rectangle), *wrist width* (i.e., the longest side of the circumscribed rectangle) and *wrist circumference* (i.e., the measurement of the perimeter by wrapping a tape measure around the wrist), respectively): (a) for men, *wt* = 43 mm, *ww* = 63.1 mm, *wc* = 172.4 mm, and (b) for women, *wt* = 37 mm, *ww* = 56.1 mm, *wc* = 149.5 mm. Moreover, for defining an acceptable value of the WP’s length, the mean values of the human forearm in healthy people should be considered, since a WP must be inserted without altering the total length of the limb and by taking into account the residual part of the amputated forearm. The mean values of the forearm length, reported in the literature, are summarized in [24] as follows (*rsl* stands for *radial-stylion length*): (a) for men, *rsl* = 268.6 mm, and (b) for women, *rsl* = 242.5 mm.

The above-mentioned data allow the definition of the reference values of Table 2 for the performances of a WP and of Table 3 for the weight and dimensions of a WP.

After defining the reference design requirements for a WP, its design can start. The design procedure aims to build a device that satisfies the reference requirements summarized in Table 2 and Table 3 and implements the following steps in order: (i) identification of the most promising mechanism architectures, (ii) dimensional synthesis of the architectures identified in the previous step, (iii) evaluation and comparison of the kinetostatic performances, (iv) component design, and (v) control system design (only for active WPs).

This paper focuses on steps (i), (ii) and (iii). In the following part of this section, step (i) will be addressed together with the methodology to implement step (ii) and (iii); then, Section 3 will implement steps (ii) and (iii) with the methodology presented in this section.

### 2.1. Identification of Promising WP Architectures (Step (i))

The mechanism architectures suitable for WPs must be sought among those of spherical and quasi-spherical mechanisms, since the human wrist performs quasi-spherical motion. The orientation workspace of the terminal device (TD), which is the output link of the WP (i.e., the one that carries the artificial hand), is the reference datum to consider for selecting such architectures. The third column of Table 2 gives this design requirement in terms of RoM. The analysis of this column reveals that FE (105°) and RU (70°) have a limited mobility, whereas PS has an ample RoM (145°).

Mechanisms can be [4] *serial* or *parallel* or *hybrid* according to how the TD is connected to the frame (base). In serial mechanisms (SMs), only one open kinematic chain (i.e., constituted of binary links connected in series) connects the TD to the base; differently, in parallel mechanisms (PMs), the TD and the base are simultaneously connected by more than one open kinematic chains (limbs), which act in parallel. Eventually, in hybrid mechanisms (HMs), the connection between the TD and the base consists of one SM in series with one PM. Roughly speaking, SMs feature larger workspaces, heavier mobile masses and lower stiffness/accuracy than PMs.

The SMs of spherical/quasi-spherical mechanisms are all (actually or reducible-to) of RRR type: that is, they are constituted by three revolute (R) pairs that, in series, connect four links: the base and the TD plus two intermediate links. If the axes of the three R pairs share a common point (spherical motion center), the mechanism is spherical; otherwise, it is quasi-spherical provided that the distances among the three axes be small. This topology provides an ample orientation workspace. Nevertheless, it is not able to match the tight requirements on the overall size and weight of WPs when also the actuators must be introduced. Therefore, it is used in robotic wrists and in passive WPs.

There is a vast literature on the kinematics of spherical mechanisms [33], which includes works on the synthesis of spherical PMs [34], [35] (Vol. 4), [36,37,38,39,40,41] that list many types of PMs. Indeed, differently from SMs, there are many PM topologies able to make the TD perform spherical motion.

Some background concepts and definitions are necessary to better understand PMs’ characteristics. By definition, the connectivity of a limb is the DOF number the TD would have if it was connected to the base only through that limb. In addition, let the limb’s workspace be the workspace the TD would have if it was connected to the base only through that limb. The PM workspace is the intersection of the workspaces of all its limbs, which justifies the smaller workspace of PMs when compared to SMs.

**Figure 2:**
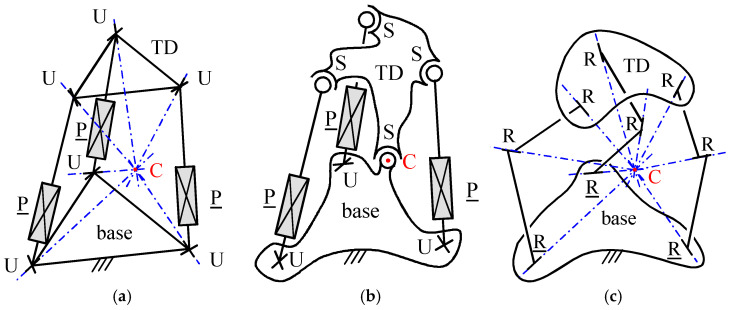
Example of SPMs (U, P, and S stand for universal joint, prismatic pair and spherical pair, respectively; the underscore indicates the actuated joint; point C is the spherical motion center): (**a**) 3UPU wrist [42], (**b**) S-3UPS wrist [43] (also named fully-parallel wrist), and (**c**) 3RRR wrist [44,45].

Consequently, a three-DOF spherical PM (SPM) is obtainable by connecting the TD to the base through a number of limbs whose workspaces share only a continuous set of TD orientations that are all reachable through spherical motions with the same center. In this context, if two or more (all the) limbs generate kinematic constraints on the TD motion that are dependent (independent), the PM is overconstrained (non-overconstrained). For instance, non-overconstrained three-DOF SPMs are obtainable by using three limbs with connectivity 5 (Figure 2a) or one limb with connectivity 3 plus three (or more) limbs with connectivity 6 (Figure 2b). Also, SPMs with more than one limb with connectivity 3 are all overconstrained (Figure 2c).

In overconstrained mechanisms (recall that mechanisms, by definition, are mechanical systems with DOFs greater than zero (i.e., with non-null mobility)), the particular geometry of the links makes the kinematic constraints dependent and the motion possible. Consequently, the presence of geometric errors makes them structures (which, by definition, are mechanical systems with zero DOF (null mobility)) since it breaks the constraints’ dependency. Differently, in non-overconstrained mechanisms, the DOF number does not depend on the links’ geometry; therefore, the presence of geometric errors does not affect it, whereas it does affect the motion type and the positioning precision. These features suggest the adoption of non-overconstrained SPMs whenever possible.

The reduced workspace of SPMs, which is due to their multi-limb architectures, suggests that obtaining all the RoMs reported in Table 3 with one 3-DOF SPM could be difficult. Indeed, an (anthropomorphic) hybrid architecture composed by one R pair (rotator) that provides the PS DOF by moving the base of a 2-DOF SPM, with respect to the forearm, that provides the remaining two DOFs (i.e., RU and FE) sounds more appropriate for a WP. This design choice is suggested in [24,46] after comparing the performances of eight wrist mechanisms relevant in the literature.

The two-DOF SPMs are also named parallel pointing systems (PPSs) [47] since they are able to control the direction of a line in the space. They enter in a number of relevant applications (e.g., orientation of antennas, telescopes, solar panels, cameras, etc.) and have been extensively studied (see [36,41,47] for instance and further references). In the context of WPs, they can be used either as stand-alone 2-DOF WPs or in combination with a rotator [28] to obtain a 3-DOF WP as explained above. The wrist DOFs they mimic change in the two applications. Indeed, a stand-alone 2-DOF WPs usually tries to mimic FE and PS, whereas, in a 3-DOF WP, the 2-DOF SPM mimics RU and FE. Since the reference RoM (Table 2) varies with the mimicked DOF, the design choices vary in the two different applications even when the adopted PPS architecture is the same.

**Figure 3:**
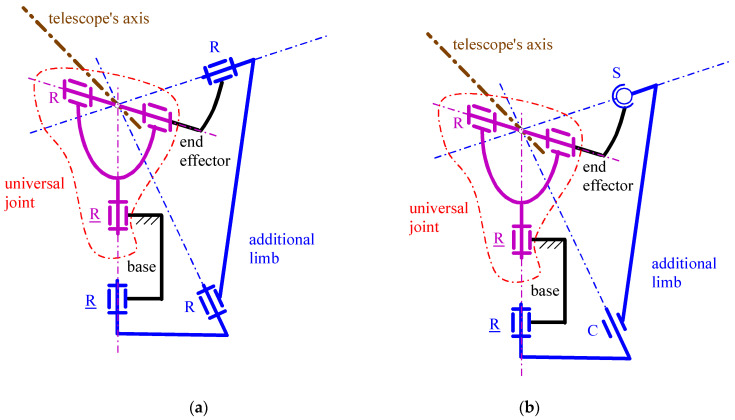
Spherical five-bar PPS of type (reproduced from [47]; C stands for cylindrical pair; the underscore denotes the actuated joint; ⊥ (·) indicates that the axes of the two adjacent joints are perpendicular to (intersect) one another): (**a**) R⊥R-R·R·R (overconstrained solution), and (**b**) R⊥R-R·CS (non-overconstrained solution with the same kinematics of (**a**)).

**Figure 4:**
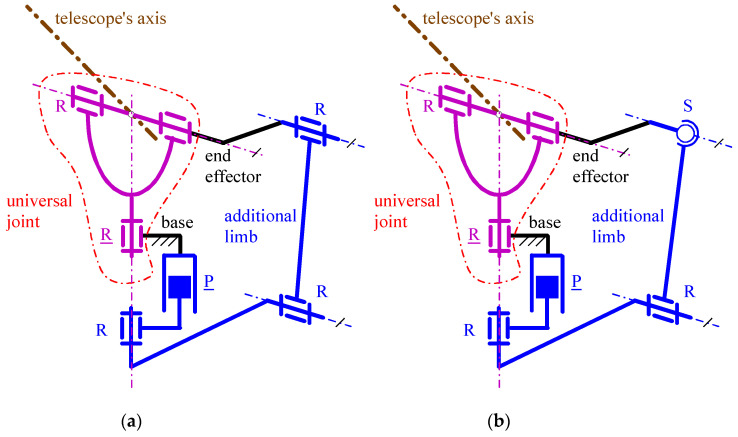
Six-bar PPS of type (reproduced from [47]; the underscore denotes the actuated joint; || (⊥) indicates that the axes of the two adjacent joints are parallel (perpendicular) to one another): (**a**) R⊥R-P||R⊥R||R (overconstrained solution), and (**b**) R⊥R-P||R⊥RS (non-overconstrained solution with the same kinematics of (**a**)).

**Figure 5:**
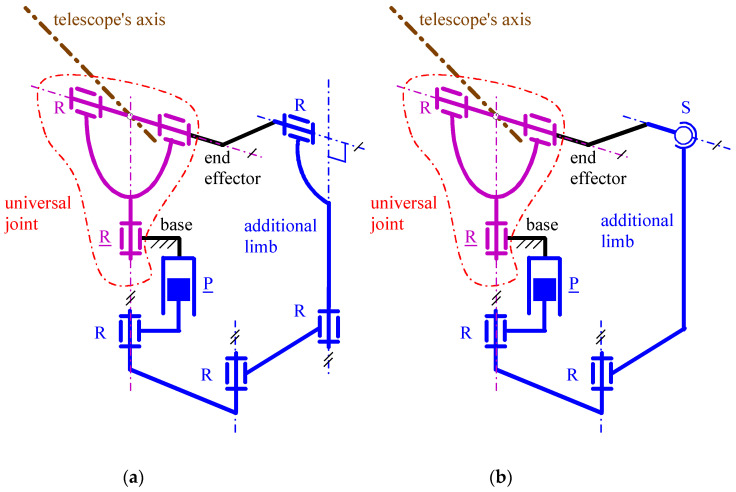
Overconstrained 7-bar PPS of type R⊥R-P||R||R||R⊥R (**a**) and its equivalent non-overconstrained 6-bar PPS of type R⊥R-P||R||RS (**b**) (reproduced from [47]; the underscore denotes the actuated joint; || (⊥) indicates that the axes of the two adjacent joints are parallel (perpendicular) to one another).

In [47], firstly, a comparative analysis of the PPSs presented in the literature brought the authors to select the six architectures (three overconstrained plus three equivalent (i.e., with the same kinematics) non-overconstrained) shown in Figure 3, Figure 4 and Figure 5 for orientating the axis of a telescope. Then, the conditions in which the end effector, which is fixed to the telescope, can perform a full rotation (i.e., RoM of 360°) around a vertical axis and a partial rotation with a RoM of 120° around an horizontal axis made the authors able to address their dimensional synthesis. All these architectures connect the end effector to the base through one U joint and an additional limb that act in parallel, thus forming a single loop.

They differ from one another regarding the type of additional limb. In particular, in Figure 3a (Figure 3b), the additional limb is a spherical R·R·R chain (R·CS chain) where the underscore denotes the actuated joint, (·) indicates that the axes of the two adjacent joints intersect one another and C stands for cylindrical pair. In Figure 4a (Figure 4b), it is a P||R⊥R||R chain (P||R⊥RS chain) where || (⊥) indicates that the axes of the two adjacent joints are parallel (perpendicular) to one another. Finally, in Figure 5a (Figure 5b), it is a P||R||R||R⊥R chain (P||R||RS chain).

**Figure 6:**
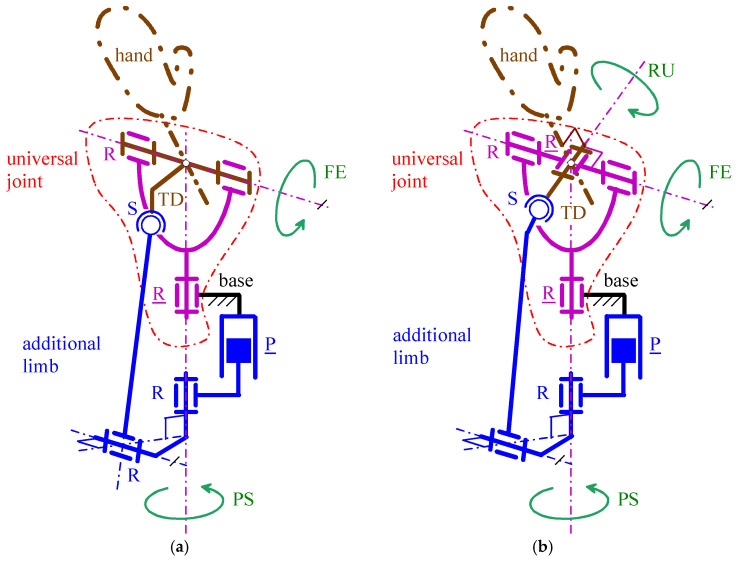
WP architectures based on the PPS architecture of Figure 4b: (**a**) 2-DOF arrangement of R⊥R-P||R⊥RS type, and (**b**) 3-DOF architecture of R⊥R⊥R-P||R⊥RS type obtained by adding one R pair.

**Figure 7:**
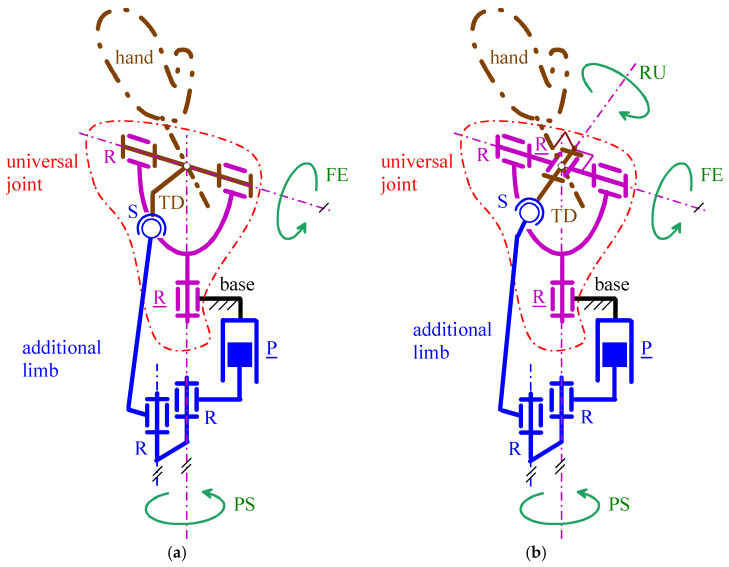
WP architectures based on the PPS architecture of Figure 5b: (**a**) 2-DOF arrangement of R⊥R-P||R||RS type, and (**b**) 3-DOF architecture of R⊥R⊥R-P||R||RS type obtained by adding one R pair.

If the horizontal (vertical) rotation axis of these PPSs coincided with the axis of a flexor (of the forearm), these architectures would become possible choices for a 2-DOF WP mimicking FE and PS with RoM values that are greater than those reported in Table 2. In this application, the fact that the reference RoM of PS (i.e., 145° (see Table 2)) is not a full rotation is exploitable to reduce the overall sizes of the WP. From the point of view of the WP sizes’ reduction, the most promising architectures are those of Figure 4 and Figure 5. Indeed, in the architectures of Figure 3, the condition in which the axes of all the R pairs must share a common intersection practically forbids a substantial size reduction. Eventually, the preference accorded to non-overconstrained architectures brings one to identify the architectures of Figure 4b and Figure 5b as the best choices. In a stand-alone 2-DOF WP, the dimensional synthesis reported in [47] for these two architectures still holds, but some geometric parameters that do not affect their kinetostatic performances change for trying to satisfy the dimensional requirement of Table 2. Figure 6a and Figure 7a show how the architectures of Figure 4b and Figure 5b, respectively, should be modified to become compact enough for being a 2-DOF WP. The so-obtained single-looped 2-DOF WP has both the actuators on the base with the actuated P pair that controls only the FE motion and the actuated R pair that controls only the PS motion: that is, their kinematics is *fully decoupled* [47]. Decoupled kinematics simplifies the control algorithms of active WPs, and it is ideal for passive WPs where the user directly manages the WP configuration.

Conversely, if either of these two architectures were used in series with a rotator to create a 3-DOF WP, both would need to be completely redesigned, as the mimicked DOFs differ in this case (i.e., RU and FE). Nevertheless, these two architectures can be modified to include a third actuator that controls an additional R pair in a single-loop 3-DOF arrangement, thereby adding RU motion to the existing PS and FE motions. Figure 6b and Figure 7b illustrate these two novel 3-DOF WP architectures.

With reference to Figure 6b (Figure 7b), the new architecture is of R⊥R⊥R-P||R⊥RS (of R⊥R⊥R-P||R||RS) type. In this architecture, the added R pair is in series with the U joint, it is actuated and it connects the first limb to the TD. Also, the axis of this R pair intersects the axis of the second R pair of the U joint at the U joint center and is perpendicular to the axis of the second R pair of the U joint. This geometric condition makes it coincide with the rotation axis of the RU motion. Eventually, the same axis passes through the center of the S pair. This other geometric condition is necessary to decouple the TD rotation around that axis from the motion of the remaining part of the mechanism.

Therefore, also the new 3-DOF WP architectures are fully decoupled: the actuated P pair adjacent to the base controls only the FE motion, the actuated R pair adjacent to the base controls only the PS motion, and the actuated R pair adjacent to the TD controls only the RU motion. In these architectures, there are two actuators on the base and one on the TD. It is worth noting that in WPs, locating an actuator on the TD is possible, since it can be fixed on the back of the artificial hand through a casing that contains the actuator and is also usable for fixing different types of artificial hands to the TD (i.e., the casing becomes some sort of general purpose mechanical coupling).

The conclusion of step (i) is that the 2-DOF and 3-DOF WP architectures shown in Figure 6 and Figure 7 are the promising ones that must be analyzed/dimensioned in the successive design steps. They are non-overconstrained, provide RoM values greater than those of the reference ones of Table 2 and have fully decoupled kinematics.

### 2.2. Methods for Dimensional Synthesis (Step (ii)) and Performance Evaluation (Step (iii))

The dimensional synthesis (step (ii)) of the selected architectures aims to determine the links’ geometric constants that directly affect the TD motion. Therefore, in the case under study, it reduces itself to the computation of those geometric constants through the imposition of the reference RoM values reported in Table 2 and of the reference overall sizes reported in Table 3.

Regarding the performance evaluation (step (iii)), during the dimensional synthesis, parameters such as the transmission angle must be assessed and utilized to ensure satisfactory static performance. Subsequently, the JS and JT values reported in Table 2 are used to determine the required type of actuation and to evaluate the feasibility of the designed device.

This two-step procedure will be implemented in the next section.

## 3. Results

Although the selected WP architectures are derived from those shown in Figure 4b and Figure 5b [47], the dimensional synthesis of these 2-DOF architectures that was presented in [47] refers to another application with different dimensional constraints, and it is not directly applicable to the present case. Consequently, all the computations must be repeated/integrated by taking into account the values reported in Table 2 and Table 3. In the following part of this section, they will be repeated/integrated, and the kinetostatics of the sized WPs will be analyzed and evaluated.

### 3.1. Dimensional Synthesis of the Selected WP Architectures (Step (ii))

**Figure 8:**
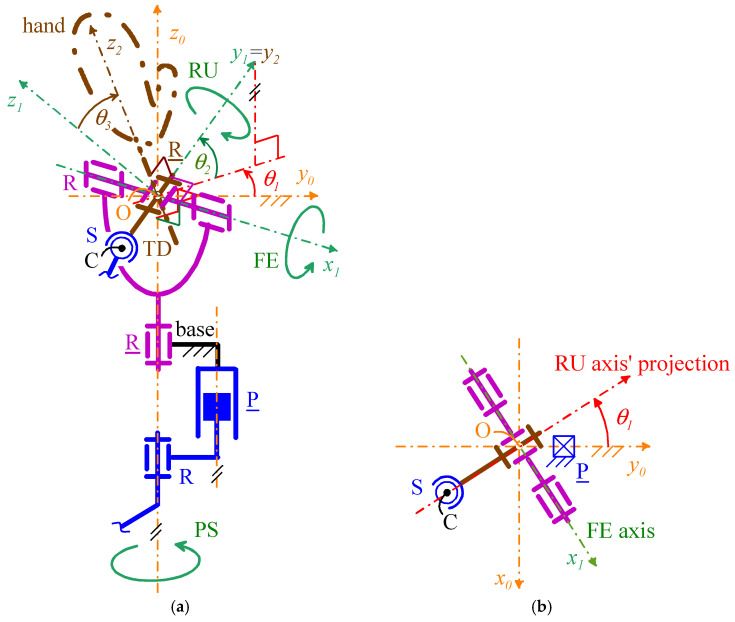
Adopted notations: (**a**) three-dimensional view of the mechanism parts that are common to all the four selected WP architectures (Figure 6 and Figure 7), and (**b**) top view of the same mechanism parts.

Figure 8 shows the adopted notations, which are valid for all the four selected WP architectures. With reference to Figure 8,

- Point *O* (point C) is the U joint (S pair) center;
- *Ox*_0_*y*_0_*z*_0_ is the Cartesian reference system, fixed to the base, with origin at *O*, *z*_0_ axis coincident with the PS rotation axis, and *y*_0_ axis that intersects the joint axis of the P pair and points from the PS rotation axis to the P-pair’s joint axis;
- *Ox*_1_*y*_1_*z*_1_ is the Cartesian reference system, fixed to the output link of the U joint, with origin at *O*, *x*_1_ axis coincident with the FE rotation axis, pointing from the ulna to the radius, and *y*_1_ axis coincident with the RU rotation axis, pointing toward the back of the artificial hand;
- *Ox*_2_*y*_2_*z*_2_ is the Cartesian reference system, fixed to the artificial hand, with origin at *O*, *y*_2_ axis coincident with the *y*_1_ axis, and *z*_2_ axis, that is the hand axis, distally directed, to use as reference when measuring the RU rotation (see Figure 1);
- θ_1_ is the joint variable of the actuated R pair adjacent to the base that also measures the PS rotation; with reference to Figure 1, positive (negative) values of θ_1_ correspond to a supination (a pronation);
- θ_2_ is the joint variable of the passive R pair of the U joint that also measures the FE rotation; with reference to Figure 1, positive (negative) values of θ_2_ correspond to a flexion (an extension);
- θ_3_ is the joint variable of the actuated R pair adjacent to the TD that also measures the RU rotation; with reference to Figure 1, positive (negative) values of θ_3_ correspond to a radial deviation (an ulnar deviation). In 2-DOF WP, θ_3_ is constant and equal to 0°.

Moreover, let **i**_n_, **j**_n_, and **k**_n_ be the unit vectors of the coordinate axes *x_n_*, *y_n_*, and *z_n_*, respectively, for *n* = 0, 1, 2; the adopted notations yield the following vector relationships (see Figure 8a)

(1a) $$\left\{\begin{array}{l} i_{1}=i_{0}\cos\theta_{1}+j_{0}\sin\theta_{1}\\ j_{1}=(j_{0}\cos\theta_{1}-i_{0}\sin\theta_{1})\cos\theta_{2}+k_{0}\sin\theta_{2}\\ k_{1}=(i_{0}\sin\theta_{1}-j_{0}\cos\theta_{1})\sin\theta_{2}+k_{0}\cos\theta_{2} \end{array}\right.$$

(1b) $$\left\{\begin{array}{l} i_{2}=i_{1}\cos\theta_{3}-k_{1}\sin\theta_{3}\\ j_{2}=j_{1}\\ k_{2}=i_{1}\sin\theta_{3}+k_{1}\cos\theta_{3} \end{array}\right.$$

which, by replacing expressions (1a) into Equation (1b), gives the following explicit expression of the rotation matrix, ^0^**R**_2_ (=[^0^**i**_2_ ^0^**j**_2_ ^0^**k**_2_], where ^n^(·) denotes (·) when measured in *Ox_n_y_n_z_n_*) transforms vector components measured in *Ox*_2_*y*_2_*z*_2_ into vector components measured in *Ox*_0_*y*_0_*z*_0_

(2) $$R_{2}=\left[\begin{array}{ccc} \cos\theta_{1}\cos\theta_{3}-\sin\theta_{1}\sin\theta_{2}\sin\theta_{3}, & -\sin\theta_{1}\cos\theta_{2}, & \cos\theta_{1}\sin\theta_{3}+\sin\theta_{1}\sin\theta_{2}\cos\theta_{3}\\ \sin\theta_{1}\cos\theta_{3}+\cos\theta_{1}\sin\theta_{2}\sin\theta_{3}, & \;\;\cos\theta_{1}\cos\theta_{2}, & \sin\theta_{1}\sin\theta_{3}-\cos\theta_{1}\sin\theta_{2}\cos\theta_{3}\\ -\cos\theta_{2}\sin\theta_{3}, & \sin\theta_{2}, & \cos\theta_{2}\cos\theta_{3} \end{array}\right]$$

**Figure 9:**
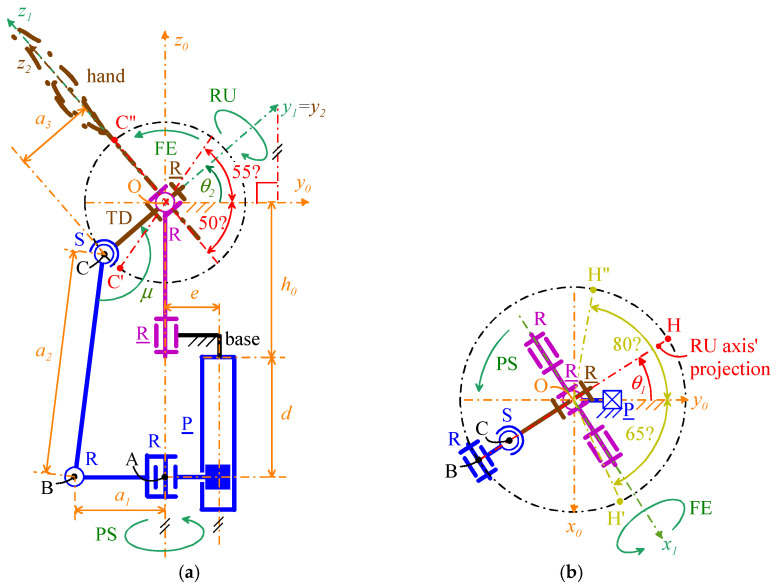
Definition of the geometric parameters of the mechanisms shown in Figure 6: (**a**) projection of a configuration with θ_1_ = θ_3_ = 0° onto the meridian plane passing through the PS rotation axis for the mechanism of Figure 6b, and (**b**) top view of the same mechanism at a configuration with θ_1_ ≠ 0°.

**Figure 10:**
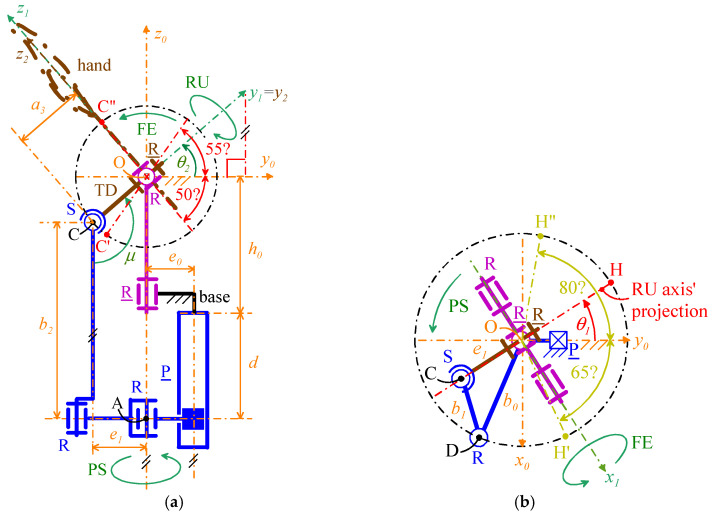
Definition of the geometric parameters of the mechanisms shown in Figure 7: (**a**) projection of a configuration with θ_1_ = θ_3_ = 0° onto the meridian plane passing through the PS rotation axis for the mechanism of Figure 7b, and (**b**) top view of the same mechanism at a configuration with θ_1_ ≠ 0°.

**Figure 11:**
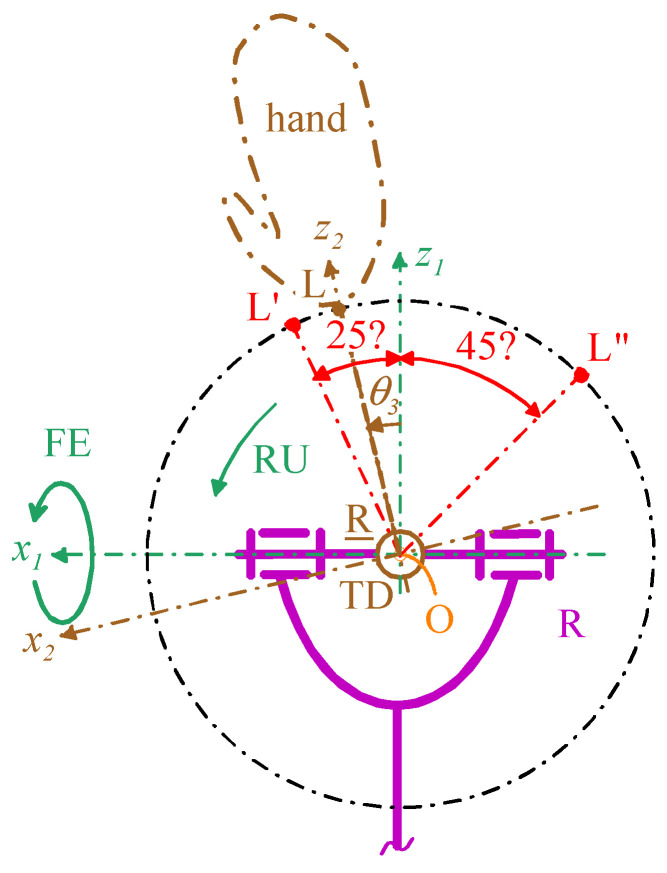
Projection of TD, U joint wishbone, and U joint output link onto the plane passing through the FE axis and perpendicular to the RU rotation axis for a mechanism configuration with θ_1_ = θ_2_ = 0°.

With reference to Figure 6 and Figure 7, in all the four architectures, the decoupling between PS and FE motions comes from the coincidence of the axis (PS rotation axis) of the actuated R pair adjacent to the base and the axis of the passive R pair adjacent to the P pair. Consequently, the FE analysis is conducible on a planar scheme obtained by projecting the mechanism onto the meridian plane passing through the PS axis and perpendicular to the FE axis (Figure 9a and Figure 10a). Moreover, the PS analysis is conducible on a planar scheme obtained by projecting the mechanism on a plane perpendicular to the PS axis (Figure 9b and Figure 10b). Eventually, in the novel 3-DOF architectures (Figure 6b and Figure 7b), the PS and FE analyses coincide with those of the 2-DOF architectures, and the RU analysis is conducible by projecting the TD together with the U joint onto the plane passing through the FE axis and perpendicular to the RU rotation axis (Figure 11).

The angle θ_1_ and θ_3_ are at same time actuated joint variables and orientation parameters of the TD. Therefore, the actuators of the joints related to them directly (and independently) control two output motion parameters. Differently, the relationship between θ_2_ and the actuated P pair’s joint variable that controls it must be determined. Such a relationship does not depend on the values of the other two actuated-joint variables; therefore, without losing generality, it can be deduced by considering a mechanism configuration with θ_1_ = θ_3_ = 0°. Figure 9a (Figure 10a) shows the projection of such a configuration onto the meridian plane passing through the PS rotation axis for the mechanism of Figure 6b (of Figure 7b). In this figure, *d* is the joint variable of the actuated P pair.

For the mechanisms of Figure 6, the analysis of Figure 9a reveals that the following relationship holds (the definition of the geometric parameters μ, *a*_1_, *a*_2_, *a_3_*, and *h*_0_ is in Figure 9a):

(3) $$\left.\begin{array}{l} a_{2}\sin(\mu -90{}^{\circ}-\theta_{2})=a_{1}-a_{3}\cos\theta_{2}\\ a_{2}\cos(\mu -90{}^{\circ}-\theta_{2})=h_{0}+d-a_{3}\sin\theta_{2} \end{array}\right\}\Rightarrow a_{2}^{2}=a_{1}^{2}+{\left(h_{0}+d\right)}^{2}+a_{3}^{2}-2a_{3}[a_{1}\cos\theta_{2}+(h_{0}+d)\sin\theta_{2}]$$

which, through the substitutions $\sin\theta_{2}=2t_{2}/(1+t_{2}^{2})$ and $\cos\theta_{2}=(1-t_{2}^{2})/(1+t_{2}^{2})$ where $t_{2}=\tan(\theta_{2}/2)$, becomes a quadratic equation in *t*_2_. This equation is solvable in closed form and gives the following explicit expression of $t_{2}=\tan(\theta_{2}/2)$ as a function of the actuated-joint variable *d*:

(4) $$t_{2,i}=\frac{-p_{1}+{\left(-1\right)}^{i}\sqrt{{p_{1}}^{2}+{p_{2}}^{2}-{p_{3}}^{2}}}{p_{3}-p_{2}}\;\;\;\;\;i=0,1$$

where $p_{1}=-2a_{3}(h_{0}+d)$, $p_{2}=-2a_{3}a_{1}$, and $p_{3}=a_{1}^{2}+{\left(h_{0}+d\right)}^{2}+a_{3}^{2}-a_{2}^{2}$. Since −90° < θ_2_ < 90° in the case under study, only one out of the two solutions provided by Formula (4) is valid.

For the mechanisms of Figure 7, the analysis of Figure 10a reveals that the following relationships hold (the definition of the geometric parameters *b*_2_, *a*_3_, *e*_1_, and *h*_0_ is in Figure 10a):

(5a) $$h_{0}+d=b_{2}+a_{3}\sin\theta_{2}\Rightarrow \sin\theta_{2}=\frac{h_{0}+d-b_{2}}{a_{3}}$$

(5b) $$e_{1}=a_{3}\cos\theta_{2}$$

Equation (5a) states a one-to-one relationship between the values of θ_2_ and *d*, since −90° < θ_2_ < 90° in the case under study, and it straightforwardly allows the computation of θ_2_ as a function of *d*.

#### 3.1.1. FE Analysis

The extreme values of θ_2_ must be (see Table 2 and Figure 9a and Figure 10a) θ_2,max_ = 55° and θ_2,min_ = −50°. Also, the P-pair stroke, *s_p_*, is, by definition, computable through the following formula:

(6) $$s_{P}=d(\theta_{2,\max})-d(\theta_{2,\min})$$

where the explicit expression of the function *d*(θ_2_) depends on the considered mechanism.

For the mechanisms of Figure 6, *d*(θ_2_) is geometrically deducible from Figure 9a that provides the following relationship

(7) $$h_{0}+d=a_{3}\sin\theta_{2}+\sqrt{a_{2}^{2}-{\left(a_{1}-a_{3}\cos\theta_{2}\right)}^{2}}\Rightarrow d=a_{3}\sin\theta_{2}-h_{0}+\sqrt{a_{2}^{2}-{\left(a_{1}-a_{3}\cos\theta_{2}\right)}^{2}}$$

which yields

(8) $$s_{P}=a_{3}(\sin\theta_{2,\max}-\sin\theta_{2,\min})+\sqrt{a_{2}^{2}-{\left(a_{1}-a_{3}\cos\theta_{2,\max}\right)}^{2}}-\sqrt{a_{2}^{2}-{\left(a_{1}-a_{3}\cos\theta_{2,\min}\right)}^{2}}$$

If $\left|\theta_{2,\max}\right|=\left|\theta_{2,\min}\right|$, the two square roots on the right-hand side of Formula (8) are equal to one another and cancel themselves, thus simplifying the formula to $s_{P}=a_{3}(\sin\theta_{2,\max}-\sin\theta_{2,\min})$. If $\left|\theta_{2,\max}\right|>\left|\theta_{2,\min}\right|$ ($\left|\theta_{2,\max}\right|<\left|\theta_{2,\min}\right|$), the first (the second) square root on the right-hand side of formula (8) is greater (smaller) than the second (the first) one and $s_{P}>a_{3}(\sin\theta_{2,\max}-\sin\theta_{2,\min})$ (and $s_{P}<a_{3}(\sin\theta_{2,\max}-\sin\theta_{2,\min})$). In the case under study (i.e., $a_{1}\approx a_{3}\ll a_{2}$, θ_2,max_ = 55° and θ_2,min_ = −50°), the analysis of Figure 9a reveals that $s_{P}<2a_{3}\sin(55{}^{\circ})=1.64\;a_{3}$.

For the mechanisms of Figure 7, *d*(θ_2_) is deducible from Equation (5a) that provides the following relationship

(9) $$d=b_{2}-h_{0}+a_{3}\sin\theta_{2}$$

which yields

(10) $$s_{P}=a_{3}(\sin\theta_{2,\max}-\sin\theta_{2,\min})$$

In the case under study, Formula (10) gives $s_{P}=1.59\;a_{3}$.

Since the value of the geometric constant *a*_3_ practically coincides with half of the WP thickness (see Figure 9a and Figure 10a), the choice *a*_3_ = 20 mm will be adopted, which is consistent with the maximum value of the WP thickness, 45 mm, reported in Table 3. Such a choice brings one to compute $s_{P}=30.28$ mm for the mechanisms of Figure 6 and $s_{P}=30.18$ mm for the mechanisms of Figure 7. Accordingly, in this design, the value $s_{P}=30.3\;$ mm is chosen for all the four selected WP architectures.

The devices shown in Figure 9a and Figure 10a, which are well proportioned, suggest that the geometric constant *h*_0_ should be chosen more or less equal to *s_p_*. Accordingly, the choice $h_{0}=35\;$ mm is adopted here, which brings one to compute something less than 70 mm as the total length for the mechanisms under design. Such a value can be considered as the total length of the WP when employed as a passive WP, and since it is lower than the maximum values (80–110 mm) reported in Table 3 for this parameter, the conclusion is that a passive WP based on the proposed architectures will be compact enough.

#### 3.1.2. PS Analysis

The extreme values of θ_1_ must be (see Table 2 and Figure 9b and Figure 10b) θ_1,max_ = 80° and θ_1,min_ = −65°. The top views shown in Figure 9b and Figure 10b highlight that such extreme values are reachable by leaving an ample region, more or less coincident with the one geometrically identified by the inequality *y*_0_ > 0, that is not crossed by moving links and can be used to place the actuators.

Table 3 gives 65 mm as a reference for the maximum width of the WPs. Figure 9b and Figure 10b show that the width of the U-joint’s wishbone is the size that determines the WP width for the studied mechanisms. Consequently, this wishbone width must not exceed 65 mm in this WP design. This value is sufficient to manufacture a stiff and high-load resistant U joint that can contain all the accessories of the WP; hence, it does not constitute a tight limitation.

#### 3.1.3. RU Analysis

The RU analysis pertains to only the two 3-DOF architectures of Figure 6b and Figure 7b. In these architectures, the RU DOF is decoupled from the other two DOFs and the links—namely the TD, the U-joint wishbone, and the U-joint output link—that could restrict the RU motion are identical in both cases. Therefore, without loss of generality, this analysis can be applied to both architectures by considering only the projection of the aforementioned links onto the plane that passes through the FE axis and is perpendicular to the RU rotation axis for a mechanism configuration with θ_1_ = θ_2_ = 0°. Figure 11 illustrates this projection.

The extreme values of θ_3_ must be (see Table 2 and Figure 11) θ_3,max_ = 25° and θ_3,min_ = −45°. The analysis of Figure 11 reveals that these extremes are reachable without adopting further geometric constraints on the link sizes. In addition, the same figure highlights that a rotary actuator with the rotation axis parallel to the z_2_ coordinate axis can be easily fixed to the back of the artificial hand and, by transmitting the motion through a simple bevel gear set, can control the RU rotation in the actuated R pair.

### 3.2. Kinetostatic Analysis and Performance Evaluation of the Sized WP Architectures (Step (iii))

In the studied mechanisms (Figure 8), the joint variables θ_1_ (PS rotation) and θ_3_ (RU rotation) are directly controlled by the actuator with at most a gear train in between. Therefore, for the joints these variables refer to, reaching the requested kinetostatic performances, that is, the JS and JT values reported in Table 2, uniquely depends on the chosen actuator and gear train. Section 4 will address the actuator choice and the related issues.

Conversely, the joint variable θ_2_ (FE rotation) is indirectly controlled by the actuator through a mechanical transmission. Consequently, the kinetostatic performance of the related joint also depends on the design of the mechanical transmission. For the studied mechanisms, the FE actuation employs mechanical transmissions that function as planar mechanisms (i.e., those shown in Figure 9a and Figure 10a). In planar mechanisms, the kinetostatic performance can be evaluated by using the *transmission angle*, μ, [48] which is so defined that |μ − 90°| is the angle between the directions of the transmitted force and of the transmitted velocity whose dot product gives the transmitted mechanical power. Accordingly, the best value of μ is 90°, which minimizes the magnitude of the transmitted force at parity of transmitted power.

As a general guideline, transmission angles satisfying the condition |μ − 90°| < 40° (max 50°) [49] are considered acceptable for a well-designed device. However, for applications with reduced loads, this limitation may be relaxed. For the mechanisms under study, the transmission angle, μ, is defined in Figure 9a and Figure 10a. These considerations lead to the imposition of μ = 90° for the neutral position of the hand (i.e., when θ_2_ = 0), which is a configuration approximately at the midpoint of the FE range of motion.

For the transmission angle of the mechanisms of Figure 6, the related Figure 9a shows that the following relationship holds:

(11) $$\mu =\theta_{2}+90{}^{\circ}+\arcsin\left(\frac{a_{1}-a_{3}\cos\theta_{2}}{a_{2}}\right)$$

which gives μ = 90° at θ_2_ = 0 if and only if $a_{1}=a_{3}$, which, for the already chosen value of $a_{3}\;(=20\;\mathrm{mm})$, gives $a_{1}=20$ mm. Also, the adoption of this condition together with Equation (7) yields $a_{2}=d(0{}^{\circ})+h_{0}$ and brings one to choose $d(0{}^{\circ})=0.5\;s_{P}=15.15$ mm for minimizing the WP length. This choice gives $a_{2}=50.15$ mm for the already chosen value of $h_{0}\;(=35\;\mathrm{mm})$.

For the transmission angle of the mechanisms of Figure 7, the related Figure 10a shows that the following relationship holds:

(12) $$\mu =\theta_{2}+90{}^{\circ}$$

which always gives μ = 90° at θ_2_ = 0. Nevertheless, even in this case, the need to reduce the WP cross-section brings one to choose $b_{0}\leq a_{3}\;(=20\;\mathrm{mm})$ and *b*_1_ as small as possible (see Figure 10b). Here, the choices $b_{0}=15$ mm and $b_{1}=7$ mm are adopted. Regarding *b*_2_, Equation (9) yields $b_{2}=d(0{}^{\circ})+h_{0}$ and brings one to choose $d(0{}^{\circ})=0.5\;s_{P}=15.15$ mm for minimizing the WP length. This choice gives $b_{2}=50.15$ mm for the already chosen value of $h_{0}\;(=35\;\mathrm{mm})$.

## 4. Discussion

**Figure 12:**
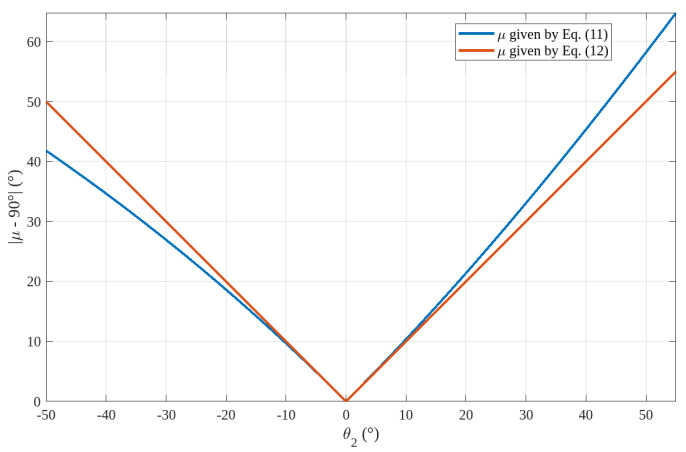
Diagrams of |μ − 90°| as a function of θ_2_.

Table 4 summarizes the selected values for the mechanism dimensions defined in Figure 9 and Figure 10. These values ensure that the transmission angle, μ, achieves its optimal value (i.e., 90°) at θ_2_ = 0, corresponding to the neutral position of the FE. However, it is important to discuss the extent of the θ₂ range in which the condition |μ − 90°| < 40° (max 50°) is satisfied, indicating a sufficiently favorable transmission angle for the hand to handle high loads effectively. Figure 12 shows the diagrams of |μ − 90°| as a function of θ_2_ both for μ given by Equation (11) (i.e., for the mechanisms of Figure 6) and for μ given by Equation (12) (i.e., for the mechanisms of Figure 7). These diagrams have the following features. For negative values of θ_2_ (i.e., in extension), all the proposed WP architectures have acceptable values of μ even though those of Figure 6 have better a kinetostastic performance. Differently, for positive values of θ_2_ (i.e., in flexion), the WP architectures of Figure 6 have acceptable performances up to θ_2_ = 43°, whereas those of Figure 7 have acceptable performances up to θ_2_ = 50° and, in general, have a slightly better behavior across the entire range. In conclusion, the kinetostatic performance degrades only near the extreme flexion angle. This behavior is similar to that of the human wrist, which gradually loses its ability to withstand external loads as it approaches the extremes of its range of motion (RoM).

Regarding the choice of the actuators, the values of JS and JT reported in Table 2 indicate actuator power requirements ranging from 16 W, for rotating an external load of 8 Nm (JT) at 2 rad/s (JS), to 52 W, for rotating an external load of 13 Nm (JT) at 4 rad/s (JS). The market offers various geared BLDC motors (i.e., brushless DC motors paired with a planetary gear set) capable of delivering up to 30 W while maintaining compact dimensions within a cylindrical volume of 20–25 mm in diameter and 80–100 mm in height. However, achieving 50 W with commercially available products of similar size and volume is significantly more challenging.

Using a 30 W geared BLDC motor would result in an operating range from rotating an external load of 15 Nm at 2 rad/s to rotating an external load of 7.5 Nm at 4 rad/s. This operating range appears to be a good compromise. Therefore, this choice will be adopted in the design.

Two of these BLDC motors must be mounted on the base with the axis parallel to the PS rotation axis (Figure 8a): one for actuating the R pair adjacent to the base and the other for actuating a ball screw that generates the linear translation of the P pair. In addition, a third one must be mounted in a casing embedded in the TD that will be also used to fix the artificial hand to the TD. Since this casing practically becomes the back of the artificial hand in the complete prosthesis (i.e., artificial hand plus WP), this mounting is possible without any risk of limiting the motion of the same prosthesis.

Regarding the sizes of the so-sized active WP, the following considerations are possible. A mini ball screw can have a cross-section inscribable in a square with a side of 18–20 mm according to the commercial products’ data. Consequently, locating side-by-side on the base two cylindrical geared BLDC motors, each with a diameter of 20–25 mm, together with a mini ball screw yields a WP cross-section with a width of 60–70 mm and thickness of 40–45 mm (20–25 mm due to the actuators and 20 mm due to *a*_3_ (see Figure 9 and Figure 10)). Such sizes practically are in the ranges defined in Table 3. Differently, for the total length of the active WP, adding the length, 80–100 mm, of the actuation package to the already determined value, 35 mm (see Table 4), of *h*_0_ (see Figure 9 and Figure 10) yields a total length of 115–135 mm. These length values exceed the reference values reported in Table 4, although they are comparable to the lengths of other WPs proposed in the literature (see [24,28] for example).

## 5. Conclusions

Reference design data for wrist prostheses (WPs) were extracted by analyzing the literature on human wrist mobility. Additionally, the potential of parallel mechanisms in WP design was explored, leading to the proposal of four novel WP architectures: two with two DOFs and two with three DOFs.

All the proposed architectures feature a single-loop topology with a reduced number of links, enabling each DOF to be controlled independently of the others. As a result, they exhibit fully decoupled kinematics. Furthermore, they can be integrated with any type of artificial hand.

The dimensional synthesis of the proposed WP architectures has demonstrated that they can meet all the mobility requirements needed for activities of daily living (ADLs) while maintaining a compact overall size when used as passive WPs. When used as active WPs, they retain a compact shape, albeit one that is slightly longer than the reference design length.

The kinetostatic analysis of these architectures has shown that their performance is comparable to that of the human wrist. Specifically, they exhibit good load-bearing capacity in the neutral pose, which diminishes as they approach the extremes of their range of motion (RoM).

Finally, the proposed architectures could also be employed in humanoid robots to mimic wrist motion in the upper limbs. This work completes the first step of a research project aiming at developing new concepts of mechatronic wrists.

## Figures and Tables

**Table 1:** Wrist mobility, joint speed (JS) and joint torque (JT) [24]: extreme values for AROM, for RoM in ADLs, for JS in ADLs, and for JT as a function of the motion type (see Figure 1).

DOF	Motion Type	AROM (°)	ADLs RoM(°)/JS(rad/s)	JT (Nm)	
				Men	Women
PS	Pronation	83	61/n.a. ^1^	9	4.5
	Supination	100	75/n.a. ^1^	9.5	4.6
FE	Flexion	76	54/1.7	12.7	8.8
	Extension	73	48/n.a. ^1^	7.9	5.8
RU	Radial Deviation	25	22/1.7	13	8.2
	Ulnar Deviation	45	38/n.a. ^1^	12.4	8

^1^ n.a. stands for “not available”.

**Table 2:** Reference requirements for the performances of a WP.

DOF	Motion Type	RoM (°)	JS (rad/s)	JT (Nm)
PS	Pronation	65	2–4	8–13
	Supination	80		
FE	Flexion	55	2–4	8–13
	Extension	50		
RU	Radial Deviation	25	2–4	8–13
	Ulnar Deviation	45		

**Table 3:** Reference values for weight and dimensions of a WP.

WP’s Parameter	Ref. Values
Weight (g)	265–370 ^1^
Thickness (mm)	35–45
Width (mm)	55–65
Length (mm)	80–110 ^2^

^1^ Values corresponding to the 25–35% of women’s mean forearm weight; ^2^ Values corresponding to the 30–45% of women’s mean forearm length.

**Table 4:** Values of the mechanism sizes defined in Figure 9 and Figure 10 that have been determined through the dimensional synthesis and the kinetostatic analysis.

Parameter	*a*_3_ (mm)	*h*_0_ (mm)	*s_p_* (mm)	*a*_2_ (mm)	*b*_2_ (mm)	*a*_1_ (mm)	*b*_1_ (mm)	*b*_0_ (mm)	Wishbone Width (mm)	Passive WP Length (mm)
Figure 9	20	35	30.3	50.15	-	20	-	-	≤65	70
Figure 10	20	35	30.3	-	50.15	-	7	15	≤65	70

## Data Availability

The original contributions presented in this study are included in the article. Further inquiries can be directed to the corresponding author.
